# Ecology of *Diaporthe eres*, the causal agent of hazelnut defects

**DOI:** 10.1371/journal.pone.0247563

**Published:** 2021-03-10

**Authors:** Roberta Arciuolo, Marco Camardo Leggieri, Giorgio Chiusa, Giuseppe Castello, Giuseppe Genova, Nicola Spigolon, Paola Battilani

**Affiliations:** 1 Department of Sustainable Crop Production, Università Cattolica del Sacro Cuore, Piacenza (PC), Italy; 2 SOREMARTEC ITALIA S.r.l., Piazzale Pietro Ferrero 1, Alba (CN), Italy; Universita degli Studi di Pisa, ITALY

## Abstract

*Diaporthe eres* has been recently reported as the causal agent of hazelnut defects, with characteristic brown spots on the kernels surface and internal fruit discoloration. Knowledge regarding the ecology of this fungus is poor but, is critical to support a rationale and effective hazelnut crop protection strategy. Therefore, a study was performed to describe and model the effect of different abiotic factors such as temperature (T, 5–35°C, step 5°C) and water activity (a_w_ 0.83–0.99, step 0.03) regimes on *D*. *eres* mycelial growth, pycnidial conidiomata development and asexual spore production during a 60-day incubation period. Alpha conidia germination was tested in the same T range and at different relative humidities (RH = 94, 97 and 100%) over 48 h incubation period. Fungal growth was observed from the first visual observation; regarding pycnidia and cirrhi, their development started after 8 and 19 days of incubation, respectively and increased over time. The optimum T for growth was 20–25°C and for pycnidia and cirrhi development was 30°C; a_w_ ≥ 0.98 was optimal for the tested steps of the fungal cycle. The best condition for conidial germination of *D*. *eres* was at 25°C with RH = 100%. Quantitative data obtained were fitted using non- linear regression functions (Bete, logistic and polynomial), which provided a very good fit of the biological process (R^2^ = 0.793–0.987). These functions could be the basis for the development of a predictive model for the infection of *D*. *eres* of hazelnuts.

## Introduction

*Diaporthe eres* was recently reported as the causal agent of hazelnut defects, which produces brown spots on the kernel surface or internal discoloration, which become visible inside the fruits after being cut in half (half-cut; [1, 2]). *D*. *eres*, was also associated, together with other *Diaporthe* spp., for causing wood cankers of fruit and nut crops in northern California [3], associated with black tip and necrotic spots on hazelnut kernles in Chile [4] and with visible mold on hazelnut in Oregon [5]. Moreover, *D*. *eres* was observed in symptomatic trunk and branches in *Corylus avellana* [6], and it is considered to be a minor pathogen of woody plants including cranberry [7], peach [8], pear [9], blueberry [10], and grapes [3, 11–13].

*Diaporthe* is the name recently recommended for use by the scientific community instead of *Phomopsis*, the asexual stage, more frequently observed [14]. *Phomopsis/ Diaporthe* spp. have been associated with several important diseases, with quite different symptoms, described both in annual and perennial plants [11, 15].

Based on the literature, *Diaporthe* spp. are considered monocyclic pathogens [16]. Thus, they only carry out a single cycle of infection for each growing season of the host crop. Species belonging to this genus are known to produce pycnidial conidiomata, suitable structures for the overwintering of the pathogen. At maturation, they produce cirrhi that include α conidia [17]. Temperatures between 5–36°C (optimum 27–29°C) are reported for spore germination of *D*. *eres* [18]. However, the effect of abiotic factors on ecology of *D*. *eres* is very scarce, although some knowledge is available in other crops like *Vitis vinifera* [19], *Prunus persica* [8], *Rubus* sp. [7] and *Juglans cinerea* [20] and forest trees [21]; furthermore, the ecology and infection cycle of *Diaporthe/Phomopsis* on different crops have only sporadically been considered, except for *P*. *viticola* and *D*. *helianthi* [17, 22].

The fungal growth, conidia sporulation and germination are influenced by several variables, such as temperature, water activity (a_w_), pH, atmosphere composition, substrate, interaction among co-occurring microorganisms and time [23]. Generally, temperature and a_w_ are considered to be the most critical abiotic factors that define the ability of fungi to grow on plants and fruits [24]. These conditions diverge among different fungal species, and even within isolates of the same species. The temperature range for fungal growth is wider of that of a_w_. In fact, fungi could growth at temperature below 0°C up to above 40°C, while the minimum a_w_ at which growth of fungi has been observed is about 0.61. The minimal a_w_ values for sporulation of fungi have been investigated for only few species, but data suggested that higher a_w_ is required for spore formation than for their germination [25].

Knowledge on fungal growth and its reproduction are crucial components for describing the infection cycle, understanding the epidemiology of pathogens, and to develop mechanistic models able to predict disease development on the crop during the growing season. Predictive models are crucial in sustainable agriculture, and they are the best support to optimize the application of control measures. The rationale control of harmful organisms for the plants is fundamental for ensuring agricultural productivity while maintaining economic and environmental sustainability; in this context, predictive tools are becoming more common and largely adopted [26].

Many predictive models have been developed and used to model the growth of different fungal species [27, 28]; however, no studies exist concerning the *D*. *eres* mycelia growth, pycnidial conidiomata development, asexual spore production and their germination to prevent defects on hazelnuts.

Information regarding this fungal pathogen, in particular its ecological needs and the infection cycle, are critical to develop sustainable and effective crop protection systems, especially in hazelnut production. Therefore, the objectives of this study were to examine the effect of different temperatures and a_w_ regimes on *D*. *eres* mycelial growth, pycnidial conidiomata development, asexual spore production and their germination. These datasets will be crucial for the development of an effective predictive model capable to predict defective hazelnuts during the growing season.

## Material and methods

### Culture media

Potato Dextrose Agar (PDA: agar, 15 g; natural potato broth obtained from potato, 200 g; dextrose, 10 g; double distilled water, 1 L) and Water Agar (WA: agar, 20 g; double distillated water, 1L) were used to perform this study. The water activity (a_w_) of PDA and WA was a_w_ = 0.99; glycerol was added to the media, according to Dallyn and Fox (20), to have a_w_ = 0.83, 0.87, 0.90, 0.93, 0.96 and 0.98 for PDA and a_w_ = 0.94 and 0.97 for WA. The accuracy of the a_w_ modifications (±0.005) was confirmed using an Aqualab LITE (version 1.3 © Decagon devices Inc., WA, USA).

### Fungal strain and inoculum preparation

A strain of *Diaporthe eres* (PH01), isolated from defective hazelnut kernels and confirmed by morphological and molecular methods as described in Battilani et al. [1], was used in this study. This strain is stored in the fungal collection of the Università Cattolica del Sacro Cuore and Micoteca of University of Minho (MUM 20.58).

*D*. *eres* PH01 was grown on WA plates and incubated at 25°C for one week with 12-hour light photoperiod to be used as an inoculum for the planned trials. Sterilized transfer tubes, 5mm Ø, were used to take plugs from the margin of the one-week WA incubated plates and transferred to the center of unmodified PDA agar media (Petri dishes, 90 mm diameter) or to those modified a_w_ conditions.

### Experiment preparation

The study considered fungal growth, conidiomata development and maturation, α conidia production and their germination in different T and a_w_ regimes. All the studies were carried out twice.

#### Fungal growth

To study the impact of temperature (T) on *D*. *eres* growth, PDA Petri dishes (a_w_ = 0.99) were centrally inoculated with *D*. *eres* WA plugs, prepared as previously described. The dishes were incubated at different T regimes, from 5 to 35°C (steps of 5°C).

Similarly, to study the impact of a_w_ regimes, PDA plates with modified a_w_ (0.83–0.99) were centrally inoculated with *D*. *eres* WA plugs and incubated at 25°C. All the replicates and treatments were incubated in the dark for 60 days.

In each replicate plate, two perpendicular colony diameters were measured every 3–4 days up to the 30^th^ day; furthermore, additional measurements were made after 45 and 60 days in the studies of the effect of T regimes. The studies were carried out with 5 replicates per treatment.

#### Occurrence of pycnidial conidiomata

The Petri plates used for the growth study were also observed for the occurrence of pycnidial conidiomata and the presence of cirrhi with the same time frames detailed previously.

To quantify the conidial production, 5 pycnidial conidiomata with visible cirrhi were randomly selected from *D*. *eres* culture plates, for each tested T, after 60 days incubation and transferred into 1.5 ml Eppendorf tube^®^ with 200 μl of double-distilled water, crushed and made up to 1 ml with double-distilled sterile water. The number of α conidia was then counted using a Bürker chamber.

#### Conidia germination

Conidia were collected from cirrhi produced by pycnidial conidiomata of *D*. *eres* colonies incubated at 25°C for 30 days. The concentration of the conidial suspension was adjusted to 10^5^ α conidia/ml using a Bürker chamber.

Ten μl of the conidial suspension were used to inoculate 5 mm Ø WA plugs, placed on a microscope glass slide; the glass slides were incubated inside 90 mm Ø Petri dishes with wet filter paper on the bottom, according to Ciliberti et al. [29]. The filter paper was wetted with distilled water, modified with the addition of glycerol for the trial at different relative humidity (RH), to obtain 94% and 97% RH [30]. Petri dishes were sealed with Parafilm^®^ and incubated from 5 to 45°C, with 5°C steps, for 48 h. The experiment was carried out with 5 replicates per treatment.

Replicates were destructively sampled after 6, 12, 24 and 48 h. The WA plugs were stained with lactophenol blue and observed using an optical microscope (Letiz labor lux D, magnification 500 x). Spores were considered germinated when the germ tube was visible. Fifty spores were observed for each combination of T × RH and incubation time, for each WA plug; spore germination was calculated as follows:

Ger(%)=(n.germinatedconidia)(n.observedαconidia)×100 (1)

### Data analysis

Statistical analyses were performed using IBM SPSS Statistics v.25 (SPSS Inc., Armonk, NY, USA, 2019). Data on fungal growth, based on the fungal culture diameter, at different T or a_w_ regimes, were considered separately for each incubation time. They were standardized (rated on the maximum value observed), to obtain growth rates on a 0–1 scale, with 0 = no growth, and 1 = maximum growth. Growth rates of *D*. *eres*, at all incubation times, were then jointly analyzed. The same approach was applied to obtain the % of α conidial germination on a scale of 1–100, at different T or RH regimes.

For pycnidial conidiomata production, they were reported based on Growing Degree Days (GDD); -the GDD was calculated as the summation of incubation T over time. The number of pycnidia counted was standardized on the maximum value observed and rated on a 0–1 scale. One-way-ANOVA for repeated measure using the Greenhouse-Geisser correction factor was applied to fungal growth (colony diameter, mm), α conidial germination (%, arcsine transformed before analysis), pycnidial conidiomata and cirrhi occurrence (number of pycnidial conidiomata per fungal colony) and α conidial production by pycnidial conidiomata (n. α conidia/pycnidium). The Tukey test was applied to highlight significant differences between means. Different non-linear regression models were fitted to the rate data to describe fungal growth, pycnidial production and α conidial germination as function of the ecological factors (T, a_w_, RH and GDD). The equation parameters were estimated applying the non-linear regression procedure of the statistical package PASW IBM SPSS Statistics v.25 (SPSS Inc., Armonk, NY, USA, 2019) which minimizes the residual sum squares using the Levenberg-Marquardt algorithm. The best model was chosen based on the adjusted R^2^ and on the minimum number of iterations required by the algorithm to converge on parameter estimates, as indicators of goodness of fit. Minitab 18 (Minitab Inc., State College, PA, USA) was used to develop the surface response contour plots of α conidial germination data, on the combinations of T × RH. For each combination, the germination rate, computed as previously described, was used as the input for data plotting.

## Results

### Fungal growth in different temperature and water activity regimes

#### Temperature

Fungal growth was observed at all the tested Ts, between 5 and 35°C, and measured as colony diameter. Temperature regimes significantly influenced fungal growth (p≤0.001); the maximum diameter was measured at 20–25°C (mean 87.1 mm) and the minimum at 35°C (14.0 mm, -84%; Table 1). The time required to fully colonize the media in the Petri plates varied between 8 days at 20 and 25°C and 45 days at 5°C. (The raw data are reported in S1 Table, https://doi.org/10.6084/m9.figshare.13168532).

**Table 1:** Analysis of variance (ANOVA) for *Diaporthe eres* growth (colony diameter; mm), pycnidial conidiomata (n. pycnidia produced/colony) and cirrhi (n. of pycnidia with cirrhi/colony) in different regimes of temperature (5–35°C, steps of 5°C), water activity = 0.99.

Factor	Colony diameter (mm)		Pycnidial conidiomata (n°)		Cirrhi (n°)	
**Temperature (°C)**	**		**		**	
5	43.3	e	0.9	c	0.0	b
10	60.9	d	13.5	b	0.0	b
15	79.7	b	18.6	b	0.0	b
20	86.9	a	§		§	
25	87.3	a	20.9	b	0.2	b
30	74.9	c	38.4	a	6.3	a
35	14.0	f	0.0	c	0.0	b

n.s.: not significant;

*p ≤ 0.05;

**p ≤ 0.01; different letters define significant differences according to the Tukey test.

^§^ Missing data: data were not included because of technical problems arose during the trial.

#### Water activity

*D*. *eres* growth was significantly affected by a_w_ regimes (p≤0.001); the optimal growth condition was at a_w_ = 0.99 (colony diameter = 85.3 mm), and this was decreased significantly at each a_w_ change, until a_w_≥0.90 (10.7mm, -87%). The maximum diameter measured at the end of incubation period for all the a_w_ treatments are presented in Table 2. (The raw data are reported in S1 Table, https://doi.org/10.6084/m9.figshare.13168532).

**Table 2:** Analysis of variance (ANOVA) for *Diaporthe eres* growth (colony diameter; mm), pycnidial conidiomata (n. pycnidia produced/colony) and cirrhi (n. of pycnidia with cirrhi/colony) in different regimes of water activity (0.83–0.99, steps of 0.03), temperature = 25°C.

Factor	Colony diameter (mm)		Pycnidial conidiomata (n°)		Cirrhi (n°)	
**Water activity (a**_**w**_)	**		**		*	
0.83	6	f	0.0	b	0.0	c
0.87	6	f	0.0	b	0.0	c
0.90	10.7	e	0.0	b	0.0	c
0.93	54.6	d	0.0	b	0.0	c
0.96	79.3	c	54.8	a	0.0	b
0.98	82.8	b	49.0	a	1.3	a
0.99	85.3	a	60.1	a	1.1	ab

n.s.: not significant;

*p ≤ 0.05;

**p ≤ 0.01; different letters define significant difference according to the Tukey test.

### Pycnidial conidiomata and cirrhi development in different temperature and water activity regimes

#### Temperature

Pycnidial conidiomata were observed at all the tested Ts, except at 35°C; cirrhi were detected only at 25°C and 30°C.

The ANOVA showed that T significantly affected (p≤0.001) pycnidial conidiomata production. Optimal T for the pycnidial conidiomata occurrence was observed at 30°C, with a mean number of pycnidial conidiomata of 38.4 in each dish. The occurrence of pycnidial conidiomata was initiated after 8 days incubation (mean = 0.6 pycnidia/colony) and increased up to the end of the trial (mean = 35.9 pycnidia/colony; the raw data are reported in S1 Table, https://doi.org/10.6084/m9.figshare.13168532).

The occurrence of cirrhi was detected after 19 days of incubation (mean number: 0.3), increasing up to the 60^th^ day (mean number: 2.9; the raw data are reported in S1 Table, https://doi.org/10.6084/m9.figshare.13168532). Furthermore, the highest occurrence of pycnidia with cirrhi, (30%) was observed at 30°C, and remained unchanged from 45 to 60 days incubation (the raw data are reported in S1 Table, https://doi.org/10.6084/m9.figshare.13168532).

The concentration of α conidia in cirrhi was assessed after 60 days incubation. At the extreme Ts tested (5 and 35°C), no conidia were found.

#### Water activity

The pycnidial conidiomata were observed at a_w_≥0.96 and cirrhi at a_w_≥0.98, while they did not develop at lower a_w_ levels. The ANOVA showed that a_w_ was a highly significant (p≤0.001) factor for pycnidial conidiomata occurrence. The optimum a_w_ for pycnidial conidiomata development was with freely available water (a_w_ = 0.99), with the mean n. pycnidial conidiomata/colony = 60.1. Their occurrence started after 15 days incubation (mean n. pycnidial conidiomata/colony = 3.8) and increased up to the end of the study (mean number = 59.1, the raw data are reported in S1 Table https://doi.org/10.6084/m9.figshare.13168532).

Regarding pycnidia showing cirrhi, they were significantly affected by a_w_ (p≤0.005). The optimum *a*_*w*_ for cirrhi production by pycnidia was recorded at a_w_ = 0.98, with 8% of pycnidia with cirrhi after 30 days incubation. The occurrence of cirrhi was initiated after 26 days incubation (mean n. pycnidial conidiomata/colony = 0.6) and increased up to the end of the study (mean n. pycnidial conidiomata/colony = 2.1; Table 2, the raw data are reported in S1 Table https://doi.org/10.6084/m9.figshare.13168532).

### Effect of temperature and relative humidity on α conidia germination

Germination of α conidia was significantly affected by both T and RH (p≤0.001). The optimum T for α conidial germination was at 25°C, with a germination rate of 42.5%. Regarding RH, the highest α conidial germination mean rate (29.1%) was at RH = 100% and decreased with RH decrease. After 6h incubation, the mean percentage of α conidia germinated was 4.4%, and germination rate increased with time, until around 32% after 48h. No germination was observed at 5 and 45°C (Table 3, the raw data are reported in S1 Table https://doi.org/10.6084/m9.figshare.13168532).

**Table 3:** Analysis of variance (ANOVA) for *Diaporthe eres* germination (α spore germination; %) in different regimes of temperature (5–35°C, steps of 5°C), relative humidity (94–100%, steps of 3%).

	GerR %	
**Temperature (°C) (A)**	**	
5	0.0	e
10	3.9	d
15	14.5	c
20	28.8	b
25	42.5	a
30	30.7	b
35	14.7	c
40	3.6	d
45	0.0	e
**Relative humidity (%) (B)**	**	
94	3.0	c
97	14.2	b
100	29.1	a
**Interactions**		
A×B	**	

n.s.: not significant;

*p ≤ 0.05;

**p ≤ 0.01; different letters define significant difference according to the Tukey test.

### Modelling *D*. *eres* growth as function of T and a_w_

**Fig 1:**
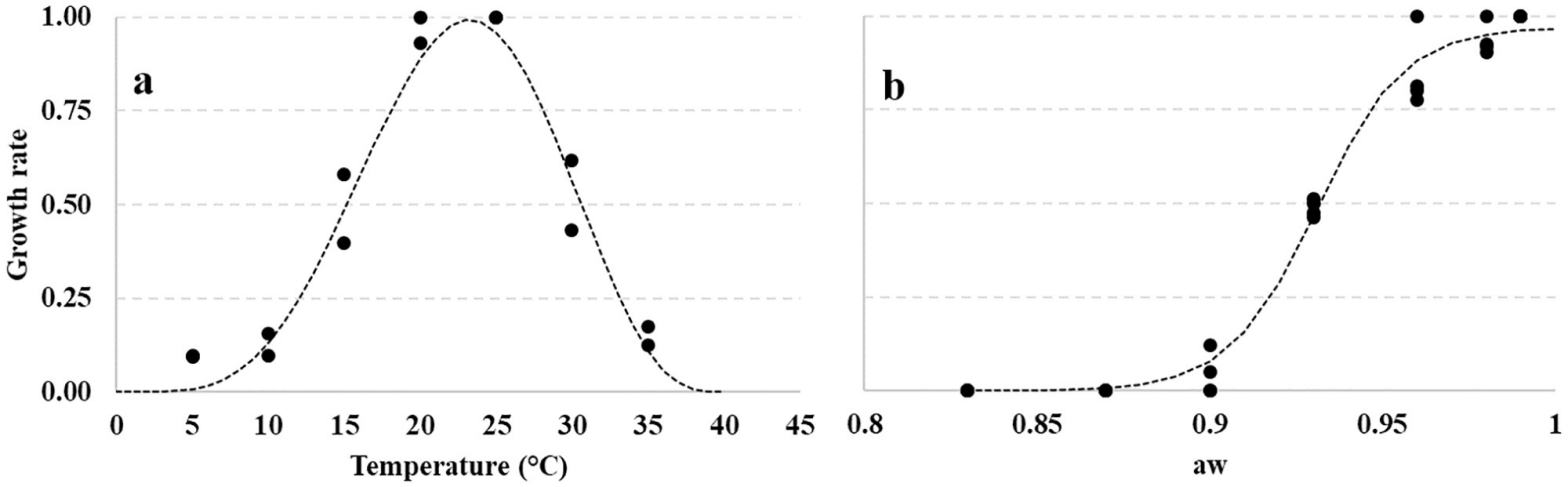
Dynamics of the growth rate of *Diaporthe eres* (a) at different T regimes (5–40°C, steps of 5°C) and (b) a_w_ (0.83–0.99, steps of 0.03). Data collected (black dots) were fitted (dotted line) by a Bete function and a logistic function, respectively (see Table 4 for equation parameters).

Two different non-linear regression models were applied to describe the quantitative relationship between fungal growth, T and a_w_ (Fig 1). Using the collected data (black dots), and excluding extreme conditions, the growth rate was computed as function of T, using the Bete equation (Eq 2) [31], and a_w_ using a logistic equation (Eq 4):

$$Y(T)=(a\times ({Teq)}^{b}\times {\left(1-Teq)\right)}^{c}$$ (2)

Where Teq is the equivalent of T, computed as:

Teq=(T-Tmin)(Tmax-Tmin) (3)

Y=c(1+exp(a+b*x)) (4)

where *a*, *b* and *c* are the estimated parameters. In Eq 3, T is the measured temperature, Tmin and Tmax are the minimum and maximum T, respectively, where growth was assumed to be absent. In Eq 4, *x* is the independent variable a_w_; in the Bete equation, *a* and *c* are the equation parameters accounting for the height and width of the bell-shaped curve, respectively, while *b* determines the T values at which the curve reaches the maximum. The estimated parameters are provided in Table 4. The adjusted R^2^ values were very good for both the parametrized factors (≥0.96, Table 4). Standard errors of parameters were lower than the parameters, confirming the goodness of fit of the applied equations.

**Table 4:** Summary of the functions used for fitting collected data at each level of the infection cycle and parameters computed.

Rate	Variable	Function	Reference in the text	Parameters			
				*A*	*B*	*C*	*R*^*2*^
Growth	T	Bete	2	5.06 *(0*.*150)*	1.39 *(0*.*050)*	3.46 *(0*.*385)*	0.964
Growth	Aw	Logistic	4	73.59 *(9*.*043)*	-79.05 *(9*.*761)*	0.97 *(0*.*022)*	0.987
Pycnidial	GDD_T	Logistic	4	6.27 *(0*.*779)*	-0.01 *(0*.*002)*	0.96 *(0*.*039)*	0.950
Pycnidial	GDD_a_w_	Logistic	4	9.64 *(1*.*855)*	-0.02 *(0*.*004)*	0.98 *(0*.*044)*	0.971
Germination	T	Bete	2	13.49 *(4*.*537)*	1.10 *(0*.*105)*	3.69 *(1*.*068)*	0.793
Germination	RH	Polynomial	5	11.85 *(1*.*967)*	1110.80 *(10*.*403)*	n.a.*	0.975

*n.a. not applicable.

Standard errors of parameters were reported in parenthesis.

### Modelling pycnidial conidiomata and cirrhi formation

**Fig 2:**
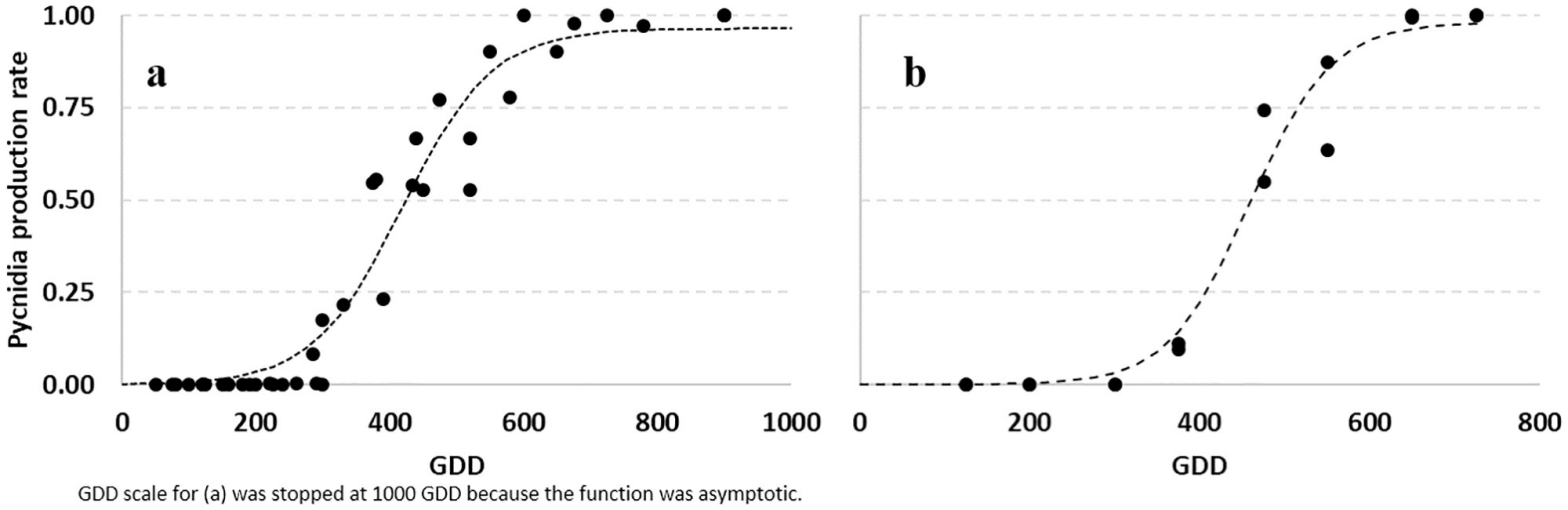
Dynamics of *Diaporthe eres* pycnidial conidiomata production rate at different growing degree days (GDD) as (a) function of T regimes and (b) a_w_. Data collected (black dots) were fitted (dotted lines) by a logistic function (see Table 4 for equation parameters).

Data on pycnidial conidiomata production were computed as a function of Growing Degree Days (GDD) both for T (Fig 2a) and a_w_ (Fig 2b); the base for GDD was considered = 0°C. The function used to fit the data (black dots) was a logistic equation (dotted lines, Eq 4). Estimated parameters are reported in Table 4.

The adjusted R^2^ for the logistic equations were 0.950 and 0.971 as a function of T and a_w_, respectively (Table 4). The standard errors of parameters were lower than the parameter, confirming the goodness of fit of the applied equations.

The production of cirrhi by the pycnidia was evaluated at different T and a_w_ regimes. It was not possible to model the collected data because the cirrhi production was observed only in 2 Ts and 2 a_w_ conditions; therefore, data were not suitable for modelling.

### Modelling *D*. *eres* germination as function of T and RH

**Fig 3:**
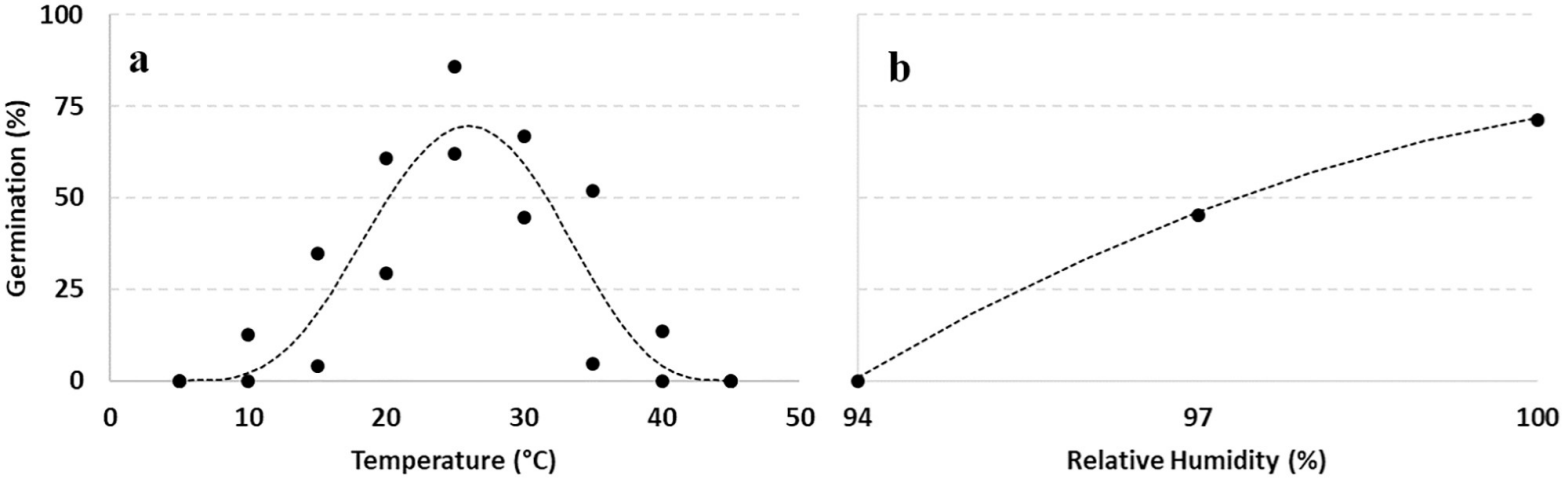
Germination of α conidia (%) at different: (a) temperatures (5–45°C, steps of 5°C) and (b) relative humidity (94–100%, steps of 3%) regimes. Data collected (black dots) were fitted (dotted lines) by a Bete and a polynomial function, respectively (see Table 4 for function parameters).

Germination of α conidia was modelled as function of T and RH (Fig 3a and 3b, respectively). Using the available data collected (black dots), the germination was computed as function of T, using the Bete equation (Eq 2, [31]), and RH using a polynomial equation (Eq 5); estimated parameters for Eqs 2 and 5 are provided in Table 4.

$$Y={a\times RH}^{2}+b\times RH+c$$ (5)

**Fig 4:**
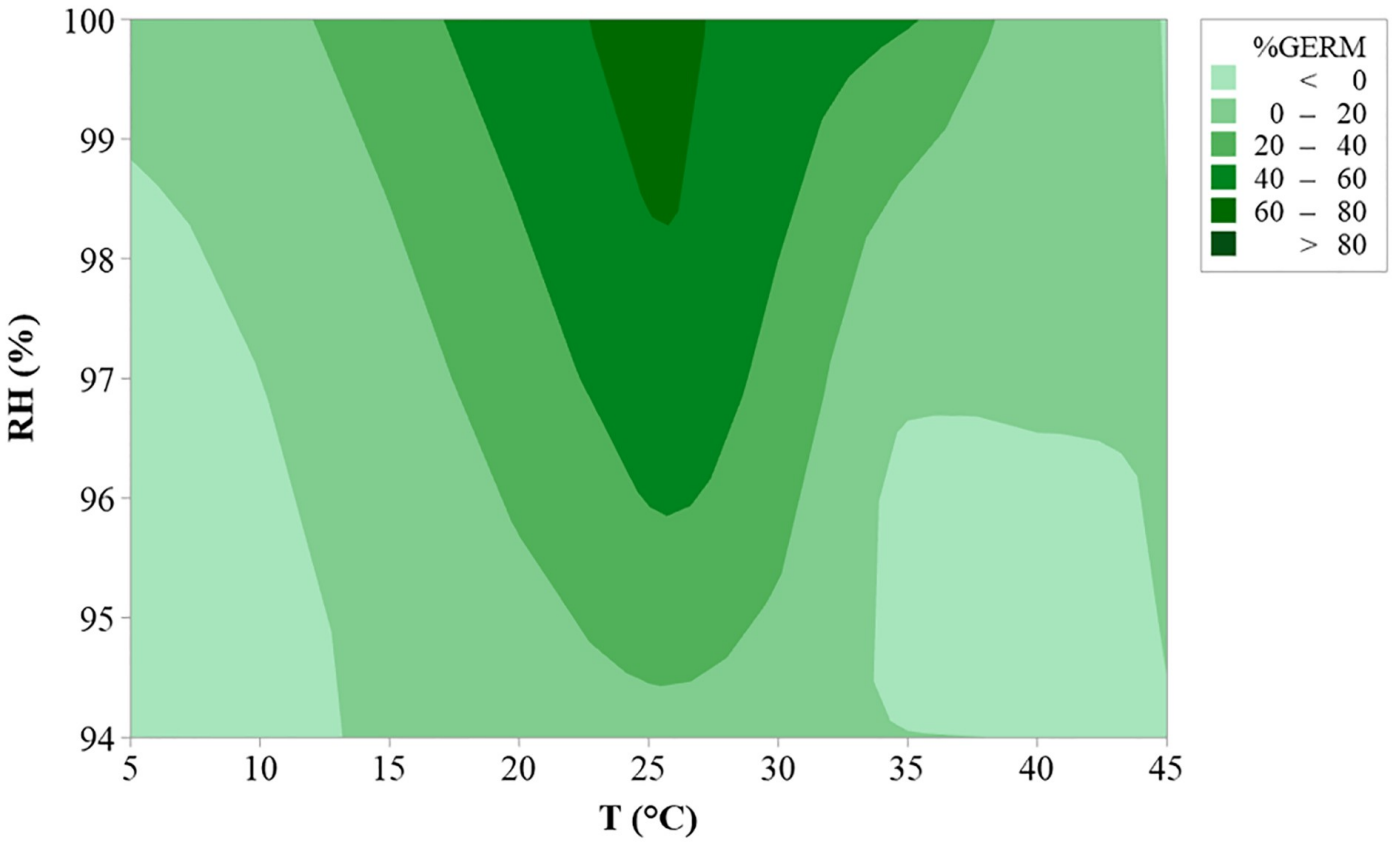
Contour plot curves of *Diapothe eres* germination (reported as the percentage of germinated α conidia; the darker the color the higher the germination) at different temperatures (5–40°C, steps of 5°C) under different relative humidity regimes (94–100%; steps of 3%).

The adjusted R^2^ for the fitted data of *D*. *eres* germination was 0.793 and 0.975, respectively for T or RH equations (Table 4). Standard errors of parameters were lower than the parameters, confirming the goodness of fit of the applied equations. The interaction between T and RH was summarized in the contour plot in Fig 4.

## Discussion

Hazelnut is a crop of global relevance; its production and the occurrence of defective kernel show fluctuations from year to year (www.fao.org; [1]), with significant economic impacts. Therefore, reducing the occurrence of defects is necessary to improve the quality of the raw nuts, save the producer income and satisfy the expanding market. *D*. *eres* was recently identified as the causal agent of hazelnut defects and neither infection cycle description nor guidelines for control measures are available. Therefore, quantitative data obtained in this study and the equations developed are very important in the approach to design and develop a mechanistic model.

It is well known that many extrinsic factors, including humidity and temperature, can affect the quality of hazelnuts. The moisture represents one of the most important factors, since a_w_ influences quality parameters, including mold if moisture is too high, shrivel if too low, color changes, and rancidity [32]. The study conducted by Mousa et al. [33] concluded that a_w_ is the deterministic factor for fungal growth and was reported that field fungi require aw>0.90 to survive [34–36]. The a_w_ measured on hazelnut changes over crop growing period with values range between 0.72 and 0.99, with risk of fungal growth in field.

The capacity of *D*. *eres* to grow and develop reproductive structures over a broad range of Ts (5–35°C) and a_w_ levels (0.83–0.99) were considered in this study for the first time. *D*. *eres* colonies grew to around 28 mm in diameter in 5 days on PDA, when the mean growth over the range of Ts was considered. No data on this fungal species are available in literature considering the same range of Ts discussed in this work. Previous studies on *Diaporthe/Phomopsis* spp. reported growth of 5–20 mm for *D*. *tanakae* and a growth > 70 mm for *P*. *mali* and *P*. *perniciosa* [37] on PDA medium at 25°C in 5 days. In the present study *D*. *eres* colonization rates in the same time frame and T conditions was 63 mm. This wide variability in relative growth between *Diaporthe* species was also found by Guarnaccia et al. [19]. They found that *D*. *bohemiae*, *D*. *celeris*, *D*. *hispaniae* and *D*. *hungariae* had variable capacity for colonization of defined media between 6 and 15 days at 21°C. However, no studies have reported intraspecific variability.

The maximum growth of *Diaporthe*/*Phomopsis* spp. was observed in a T range of 18–30°C, depending on the species. Thus, optimal T ranges between 23–30°C was reported for *Phomopsis* sp. isolated from cashew leaves [38], 25–30°C for *D*. *ueckerae*, the causal agent of dieback disease on *Michelia shiluensis* [39] and between 18–30°C for *P*. *eucommicola*, cause of canker disease in poplar [40]. However, the optimum T for mycelial growth for *P*. *vaccini* was between 20–28°C [41], at 25°C for *P*. *amygdali* [42], *P*. *cinerascens* [43] and *P*. *asparagi* [44], 28°C for *Phomopsis* sp isolated from *Eucommia ulmoides* [45] and 20°C for *P*. *destruens* [46]. A study on *D*. *eres* as the causal agent of shoot blight of peach trees in Greece reported an optimum T for mycelial growth on PDA at 25°C, although no growth was observed at 35 and 10°C after 5-day incubation periods [8]. In the present work, mycelial growth was observed at both 5 and 35°C, but with longer incubation times with an optimum at 20 and 25°C. This is similar to the strains infecting peach trees [8].

In the present study, a_w_≥0.90 was the best for *D*. *eres* growth, with a_w_≥0.96 and a_w_≥0.98 for pycnidia and cirrhi development, respectively. Previously, RH values > 95% was claimed to be necessary for *P*. *amygdali* mycelial growth, pycnidial conidiomata development, asexual spore production and their germination [42].

In this study, the number of pycnidial conidiomata counted in the fungal colonies varied at different incubation Ts, with an optimum of 0.9 pycnidia/cm^2^ of colony recorded at 30°C; pycnidia were not detected at 35°C, as also observed for *D*. *ueckerae* by Yi et al. [27]. Other studies found optimum T for pycnidial production by *Phomopsis* sp. isolated from raspberry canes and *P*. *cinerascens* between 15 and 25°C [43, 47].

The maximum number of pycnidia with cirrhi/colony and cirrhi production by *D*. *eres* was observed at 30°C in this study, with 6.3 compared to 0.2 at 25°C; no cirrhi were observed at all the other Ts and incubation times examined. In addition, a_w_ influenced cirrhi occurrence with production only observed at a_w_≥0.98. The time required for pycnidial conidiomata to produce cirrhi was around 4–5 weeks, according to Rosenberger [48] in *D*. *perniciosa* and *P*. *tanakae*. The lower and upper T limits for cirrhi development reported for *P*. *viticola* are respectively 4 and 36°C, with the optimal T at 22°C. Anco et al. [16] observed cirrhi at Ts between 18 and 25°C, after a minimum leaf wetness duration of 47 h. No previous study examined the impact of a_w_ on cirrhi production by *Diapothe/Phomopsis* spp.

The time necessary for pycnidial conidiomata and cirrhi development is quite long; indeed, diseases caused by *Diaporthe* spp are considered monocyclic [16, 49]. In the field, for apple and European pear cultures, *P*. *tanakae* developed pycnidia in May and cirrhi were observed in late May; conidia were released from the fruiting structures only during rainfall between June and September [37]. The fungus usually becomes inactive during the summer, in warm and dry conditions, but it remains active during the growing season. Rain and cold weather have a crucial role in the development of an epidemic and inoculum can be dispersed when cool and wet weather occurs [50], as also reported for *P*. *viticola* [17].

*Phomopsis/Diaporthe* produce two types of conidia: elliptical and biguttulate α conidia and long and thin β conidia; the latter were for an extended period characterized as being unable to germinate [48, 51]. More recently, germination after very long incubation time periods (144 h) was reported [52]; however, their role in infections is still unknown. Thus, since α conidia have been confirmed as the primary role as inoculum, they were the main focus in the present study.

Concentration of α conidia in cirrhi was scarcely influenced by T. However, they were strongly affected by the incubation time: in the range of Ts considered in this study, the α conidial concentration was abundant, and probably sufficiently high as a source of inoculum for the pathogen infection in the field (3.00×10^5^ α conidia/pycnidium). In fact, according to Nabetani et al. [41], from bud-break to bloom stages, during cold and wet periods, over 80% of pycnidia produced by *P*. *vaccini* contain 1–2×10^4^ conidia/pycnidium, which was considered to be a high concentration of conidia with a crucial role in disease epidemics. Moisture and recurrent precipitation events were found to promote conidial dispersal and germination, resulting in infections of the twigs, blossom blight and stem dieback of highbush blueberry.

The capacity of *D*. *eres* α conidia to germinate was observed for up to 48 h in this study, at 10–40°C at all RH conditions tested. In particular, the conidial germination started from 6 h incubation and increased during the time, from 10°C to 40°C, with the optimum at 25°C + RH = 100%; α conidial germination occurred only under high humidity conditions (RH≥94%). It was therefore confirmed the germination only over a very narrow range of RH, with a wide range of T.

Previously, *D*. *viticola* α conidia were reported to germinate in a T range of 1–37°C, with an optimum of 23°C in a few hours in free water or near 100% RH [50], with strong T x RH interaction. In fact, *D*. *viticola* α conidia germinated in 4 h at 25°C, while, at least 7–10 h with high RH conditions were necessary with 15–18°C [50].

The effects of T and a_w_ on *D*. *eres* growth were modelled using a Bete and a logistic equation, respectively. The approach has been widely described in the literature for other pathosystems, e.g., powdery mildew on cucumber [53] and maize ear rot [54], as well as for more complex situations including fungal interactions [55]. The production of pynidial conidiomata and cirrhi development were well described based on GDD as the independent variable in a logistic equation. The trend of this non-linear regression was quite similar to another S-shaped curve, the Gompertz equation [56], recently used to describe the release of *Guignardia bidwellii* conidia from overwintered grape berry mummies [57]. The fitting of non-linear functions involves considerable effort compared to polynomial equations, but the accompanying increased biological meaning [58–60] makes non-linear models the best choice to quantitatively describe biological phenomena. Finally, α conidial germination was successfully described both as a function of T and RH. This approach was previously applied to model the germination of *Aspergillus carbonarius* spores on grapes and synthetic medium [61], the germination of *Fusarium graminearum* in wheat [62] and the sporulation and germination of *F*. *langsethiae* on synthetic medium [63].

All the data fitting was successful, with very good statistical evaluation; therefore, some bricks for the future building, a mechanistic predictive model, are available and contour of suitable conditions defined. Even if based on studies managed with only one strain of *D*. *eres*, they can be assumed as representative, because intraspecies variability was never reported. There are several examples regarding the projection of quantitative data on fungal ecology in predictive models, like *Botrytis cinerea* for grapes [64] or *Fusicladium eriobotryae* in loquat [65], and that is the challenge also for *D*. *eres* in hazelnut.

However, it is important to underline that there is still a lack of knowledge on the specific pathosystem for hazelnut-*D*. *eres*. As *D*. *eres* is a monocyclic pathogen [16], it will be critical to accurately define the growth stage at which the crop is susceptible, so as the location of inoculum sources, the influence of meteorological factors, like rainfall which is assumed as critical for spore dispersal and infection [1]. Fill these gaps of knowledge would be crucial for the development of a successful mechanistic model supporting predictions of defective hazelnuts as supporting tool for the value chain stakeeholders.

## Supporting information

S1 Table (XLSX)
